# A single dose of DNA vaccine based on conserved H5N1 subtype proteins provides protection against lethal H5N1 challenge in mice pre-exposed to H1N1 influenza virus

**DOI:** 10.1186/1743-422X-7-197

**Published:** 2010-08-21

**Authors:** Haiyan Chang, Chaoyang Huang, Jian Wu, Fang Fang, Wenjie Zhang, Fuyan Wang, Ze Chen

**Affiliations:** 1 College of Life Sciences, Hunan Normal University, Changsha 410081, Hunan, China; 2 Shanghai Institute of Biological Products, Shanghai 200052, China; 3 State Key Laboratory of Virology, Wuhan Institute of Virology, Chinese Academy of Sciences, Wuhan 430071, Hubei, China; 4 Department of Immunology, Xiangya School of Medicine, Central South University, Changsha 410078, China; 5 Xinhua Hospital affiliated to Shanghai Jiaotong University of Medicine, Shanghai, 200092, China

## Abstract

**Background:**

Highly pathogenic avian influenza virus subtype H5N1 infects humans with a high fatality rate and has pandemic potential. Vaccination is the preferred approach for prevention of H5N1 infection. Seasonal influenza virus infection has been reported to provide heterosubtypic immunity against influenza A virus infection to some extend. In this study, we used a mouse model pre-exposed to an H1N1 influenza virus and evaluated the protective ability provided by a single dose of DNA vaccines encoding conserved H5N1 proteins.

**Results:**

SPF BALB/c mice were intranasally infected with A/PR8 (H1N1) virus beforehand. Six weeks later, the mice were immunized with plasmid DNA expressing H5N1 virus NP or M1, or with combination of the two plasmids. Both serum specific Ab titers and IFN-γ secretion by spleen cells in vitro were determined. Six weeks after the vaccination, the mice were challenged with a lethal dose of H5N1 influenza virus. The protective efficacy was judged by survival rate, body weight loss and residue virus titer in lungs after the challenge. The results showed that pre-exposure to H1N1 virus could offer mice partial protection against lethal H5N1 challenge and that single-dose injection with NP DNA or NP + M1 DNAs provided significantly improved protection against lethal H5N1 challenge in mice pre-exposed to H1N1 virus, as compared with those in unexposed mice.

**Conclusions:**

Pre-existing immunity against seasonal influenza viruses is useful in offering protection against H5N1 infection. DNA vaccination may be a quick and effective strategy for persons innaive to influenza A virus during H5N1 pandemic.

## Background

Human infection of highly pathogenic avian H5N1 influenza virus was first reported in Hong Kong in 1997, causing six deaths [1]. Since then, human cases of H5N1 virus infection have been continually laboratory-confirmed in many countries, with approximately 60% death rate [2]. Probable limited human-to-human spread of H5N1 subtype virus is believed to have occurred as a result of prolonged and very close contact [3]. Owing to the universal lack of pre-existing immunity to H5N1 virus in the population, pandemic caused by the virus may outbreak. Vaccination is the preferred approach for the prevention of influenza infection. Inactivated H5N1 influenza vaccines have been proved to be effective in eliciting neutralizing antibodies against the virus in clinic trials, but proved to have poor immunogenicity [4]. Novel strategies, including DNA vaccines, should be developed to cope with the H5N1 influenza virus that may cause potential pandemics.

Seasonal influenza A subtypes H1N1 and H3N2 have globally circulated in humans for a few decades. There are rare people that have no history of exposure to these viruses [5,6]. Although it is necessary to annually update vaccine strains to ensure effective protection against seasonal influenza infection in humans due to the frequent antigenic drift of the virus strains, seasonal human influenza-specific CTLs, mostly targeting conserved internal proteins, e.g., NP and M1, have been demonstrated to offer T cell cross-reactivity more or less against avian influenza H5N1 virus [6-8]. The memory T cells established by seasonal human influenza A infection could not provide adequate protection, but could alleviate symptoms of influenza H5N1 virus infection [7].

DNA vaccines based on various genes of H5N1 virus have already been explored previously, demonstrating that, when DNA vaccines encoding NP or M1 were used to immunize mice, multi-dose injection would be needed to provide effective protection [9]. In this study, a single dose of vaccination with NP, M1 or NP + M1 DNAs from A/chicken/Henan/12/2004(H5N1) virus strain was evaluated in mice pre-exposed to A/PR8(H1N1) virus, which showed that DNA vaccination might be a quick and effective strategy against H5N1 infection in individuals innaive to influenza A virus.

## Results

### Anti-H1N1 antiserum failed to afford protection against H5N1 in mice

Sera were collected and pooled from mice infected with A/PR8 (H1N1) influenza virus six weeks before. The ELISA method was used to detect the anti-H1N1 IgG Ab titers, while the HI assay to detect HI Ab titers against either H1N1 or H5N1 influenza viruses. Then 24 naive SPF BALB/c mice were passively immunized with the pooled sera by tail vein injection in a volume of 300 μl. Twenty-four hours after the serum transfer, mice were randomized into 2 groups and were challenged with a lethal dose of H1N1 and H5N1 influenza viruses, respectively. The results are shown in Table 1. High Ab titer was detected in mice after infection with A/PR8 virus. The antiserum contained high HI Ab titer against H1N1 virus but didn't contain HI Ab against H5N1 virus, as proved by the HI assay. All mice receiving serum transfer survived the lethal challenge with H1N1 virus, but none survived the lethal H5N1 challenge. The data indicated that anti-H1N1 Abs were not able to provide any protection against H5N1 influenza virus in mice.

**Table 1:** Serum Ab titers in mice exposed to A/PR8(H1N1) virus and protection offered by anti-H1N1 antiserum transfer^§^

ELISA Ab (log_2_)^a^	HI Ab (log_2_)^a^		Survival of passively immunized mice (%)	
	Anti-H1N1	Anti-H5N1	H1N1 challenge	H5N1 challenge
15.3 ± 1.15	7 ± 0*	0	100*	0

^§^Serum was collected and pooled from mice infected with A/PR8 (H1N1) influenza virus six weeks before. The IgG Ab and HI Ab titers were detected by ELISA and HI, respectively. Naive BALB/c mice were passively immunized with the pooled serum by tail vein injection in a volume of 300 μl and were then challenged with a lethal dose (20LD_50_) of H1N1 or H5N1 influenza virus after 24 hours.

^a^Values represent means ± SD of each group.

*Significant difference (*p *< 0.05), compared with the corresponding H5N1 virus group.

### Protection against H5N1 influenza virus challenge

One hundred and forty-four SPF BALB/c mice were randomized into two groups (n = 72). One group was infected with H1N1 virus, and the other was uninfected. The subsequent experimental procedure was the same for the two groups. Six weeks later, mice in each group were randomly divided into 4 subgroups (n = 18). Three subgroups were immunized with NP DNA, M1 DNA or NP + M1 DNAs, respectively, and the rest remained unimmunized as a control. Six weeks after immunization, all the mice were challenged with a lethal dose (20LD_50_) of H5N1 virus. The protective ability of DNA vaccination was determined by lung virus titer 3 days post-challenge and body weight change and survival rate of mice within 21 days.

**Figure 1:**
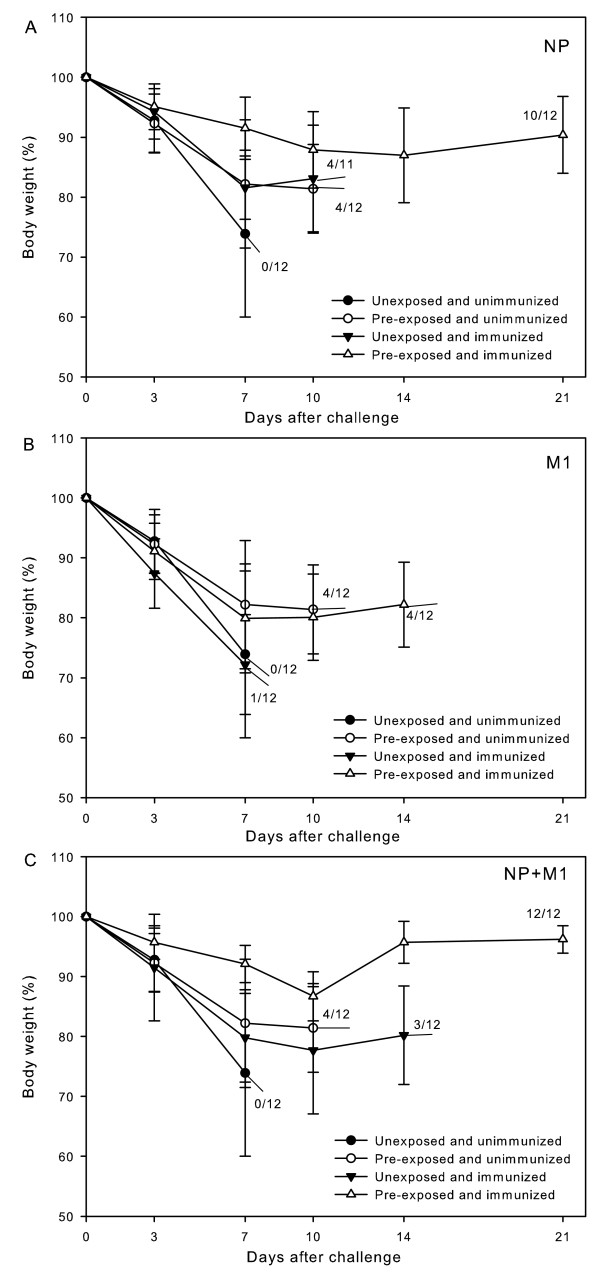
**Body weight changes of mice post-challenge**. Mice unexposed or pre-exposed to H1N1 virus were immunized with a single dose of H5N1 virus NP (A), M1 (B) or NP + M1 DNAs (C), respectively. Six weeks after immunization, all the mice were challenged with a lethal dose (20LD_50_) of H5N1 virus. Body weights of mice were recorded at 0, 3, 7, 10, 14, and 21 days after challenge.

The results are shown in Table 2 and Figure 1. For uninfected mice, a single dose of NP DNA or NP + M1 DNAs from H5N1 virus provided partial protection against homologous virus challenge, but M1 DNA seemed to have no effect, as compared with the unimmunized control. On the other hand, after infected beforehand with H1N1 virus, all the mice, including the unimmunized control, were generally provided with improved protective ability against lethal H5N1 challenge, as compared with their respective uninfected corresponding. Protection offered by NP DNA or NP + M1 DNAs was significantly better in the infected mice than in their uninfected corresponding as well as in infected but unimmunized control. One hundred percent survival rate was achieved by injection of the infected mice with NP + M1 DNAs. However, the data derived from M1 DNA vaccination were nearly the same as those from the unimmunized control in the infected group, as is the case in uninfected group.

**Table 2:** Protection provided by DNA vaccines against lethal homologous H5N1 challenge in mice unexposed and pre-exposed to H1N1 virus^§^

Group	Subgroup (DNA vaccine)	Protection against H5N1 virus challenge (20LD_50_)		
		Survival rate (survival number/total)	Body weight loss (% of the original)	Lung virus titers (log_10_TCID_50_/ml)
Unexposed to H1N1	NP DNA	4/11*	18.4 ± 1.02	9.93 ± 0.26
	M1 DNA	1/12	27.8 ± 2.86	10.25 ± 0.35
	NP+M1 DNAs	3/12	20.2 ± 0.54	8.96 ± 0.66
	Unimmunized	0/12	26.1 ± 1.76	10.85 ± 0.21

Pre-exposed to H1N1	NP DNA	10/12^a, b^	8.5 ± 2.01^a, b^	6.43 ± 0.84^a, b^
	M1 DNA	4/12	20.1 ± 2.63^a,^	10.05 ± 0.07
	NP+M1 DNAs	12/12^a, b^	7.9 ± 0.72^a, b^	7.12 ± 0.17^a, b^
	Unimmunized	4/12^a^	17.8 ± 1.29^a^	9.78 ± 1.39

^§^Mice were randomized into two groups. One group was infected with H1N1 virus, and the other was uninfected. Six weeks later, mice in each group were randomly divided into 4 subgroups. Three subgroups were immunized with a single dose of NP DNA, M1 DNA and NP+M1 DNAs, respectively, and the rest remained unimmunized as a control. Six weeks after immunization, all the mice were challenged with a lethal dose (20LD_50_) of H5N1 virus. Lung virus titers, body weight losses and survival rates of mice were determined 3 days, 7 days and 21 days post-challenge, respectively.

^a^Significant difference (*p *< 0.05), compared with the corresponding unexposed mice.

^b^Significant difference (*p *< 0.05), compared with the pre-exposed but unimmunized control.

*One mouse in the group died during anesthesia.

Some conclusions could be drawn from the above results. Pre-exposure to H1N1 virus enhanced the protective ability in mice against lethal H5N1 challenge. A single dose of H5N1 NP DNA or NP + M1 DNAs provided partial protection against lethal H5N1 virus challenge in unexposed mice and significantly enhanced protection in pre-exposed mice; however, M1 DNA was not able to provide effective protection against the virus challenge in both unexposed and pre-exposed mice.

### Ab responses

Mice were grouped and treated as described above. Three days after the lethal H5N1 challenge, six mice from each subgroup were taken out for specific IgG Ab detection, and at the same time, for lung virus titration as well, as described in the section Methods. The results are shown in Table 3. For the mice uninfected beforehand, immunization with NP DNA, M1 DNA or NP + M1 DNAs induced antigen-specific Abs. When mice were infected beforehand with H1N1 influenza virus, both anti-H5 NP and M1 Abs could be detected even in the unimmunized control mice. Injection with DNA vaccines significantly increased the specific Abs in mice pre-exposed to H1N1 virus.

**Table 3:** Specific Ab titers in unexposed and pre-exposed mice after immunization^§^

		Ab titer by ELISA (log_2_)	
Group	Subgroup (DNA vaccine)	Anti-NP	Anti-M1
Unexposed to H1N1	NP DNA	12.5 ± 1.0	Not done
	M1 DNA	Not done	10.0 ± 0.81
	NP + M1 DNAs	12.5 ± 0.58	10.5 ± 1.29

Pre-exposed to H1N1	NP DNA	22.0 ± 0.57^a, b^	Not done
	M1 DNA	Not done	12.7 ± 1.15^a, b^
	NP + M1 DNAs	22.3 ± 1.15^a, b^	13.3 ± 0.57^a, b^
	Unimmunized control	17.0 ± 1.0	9.33 ± 0.57

^§^Mice were grouped and treated as described in Table 2. Three days after the lethal H5N1 challenge, six mice from each subgroup were sacrificed for specific IgG Ab detection by ELISA. NP and M1 proteins obtained by prokaryotic expression were used to coat the microtiter plate. Values represent means ± SD of each group.

^a^Significant difference (*p *< 0.05), compared with the corresponding unexposed mice.

^b^Significant difference (*p *< 0.05), compared with the pre-exposed but unimmunized control.

### Cell-mediated immunity

**Figure 2:**
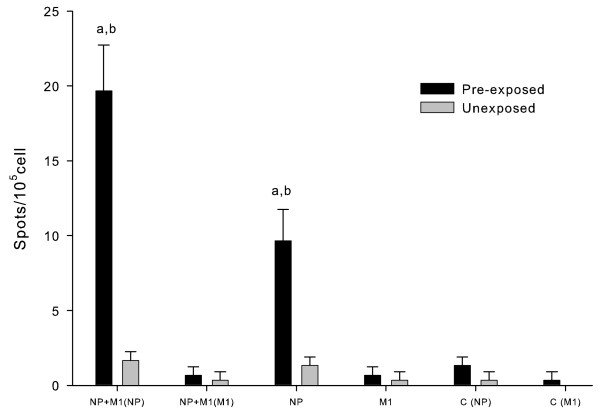
**IFN-γ secreting splenocytes in unexposed and pre-exposed mice after immunization**. BALB/c mice were randomized into two groups, one infected with H1N1 virus and the other uninfected. Six weeks later, both groups were divided into 4 subgroups. Three of the subgroups were immunized with H5 NP DNA, M1 DNA or NP + M1 DNAs, respectively, and the rest subgroup remained unimmunized. Splenocytes of mice were isolated 6 weeks after immunization and stimulated by the synthesized NP or M1 peptide. The number of IFN-γ secreting splenocytes was calculated as the average number of spots in the triplicate stimulant wells. Abscissa description: NP + M1 (NP): immunization with NP + M1 DNAs and stimulation with NP peptide; NP + M1 (M1): immunization with NP + M1 DNAs and stimulation with M1 peptide; NP: immunization with NP DNA and stimulation with NP peptide; M1: immunization with M1 DNA and stimulation with M1 peptide; C (NP): unimmunization control and stimulation with NP peptide; C (M1): unimmunization control and stimulation with M1 peptide. ^a ^Significant difference (*p *< 0.05), compared with the corresponding unexposed mice. ^b ^Significant difference (*p *< 0.05), compared with the pre-exposed but unimmunized control.

Cellular immune responses to DNA vaccines were assessed by measuring IFN-γ secretion in mouse splenocytes. BALB/c mice were randomized into two groups (n = 24). One group was infected with H1N1 virus and the other was uninfected. Six weeks later, both groups were divided into 4 subgroups (n = 6). In both groups, three of the subgroups were immunized with H5 NP DNA, M1 DNA or NP + M1 DNAs, respectively, as described above, and the rest remained unimmunized. Splenocytes of mice were isolated after 6 weeks and stimulated by the synthesized NP or M1 peptide, as described in the section Methods. The number of IFN-γ secreting splenocytes was calculated as the average number of spots in the triplicate stimulant wells. Results are shown in Figure 2. For mice uninfected beforehand, H5 NP-specific spots were a little more in mice immunized with NP DNA or NP + M1 DNAs than those in the unimmunized control, but M1-specific spots could not be clearly detected in mice immunized with M1 DNA and NP + M1 DNAs. On the other hand, for mice infected with H1N1 virus beforehand, both H5 NP- and M1- specific spots could be detected with very low number in the unimmunized control mice. The H5 NP-specific spot numbers of mice immunized with NP DNA and NP + M1 DNAs were significantly increased compared with those of the unimmunized control, and were about 5 times and 10 times, respectively, the spot numbers of the corresponding uninfected but immunized mice. M1-specific spot numbers were about equal in all immunized mice and the unimmunized control mice. To sum up, H5 NP-specific splenocytes induced by NP DNA or NP + M1 DNAs could be greatly enhanced in mice with pre-existing immunity to H1N1 virus, but H5 M1-specific splenocytes induced by M1 DNA or NP + M1 DNAs could only be slightly increased.

## Discussion

Seasonal influenza A subtypes H1N1 and H3N2, as well as type B virus, have globally co-circulated in the human population for a few decades. Infection of influenza virus induces specific immune responses in the human body, including both humoral and cell-mediated immune responses. Due to antigenic drift that is a continuous ongoing process in type A influenza virus, the immunity induced by a certain strain is usually limited. However, more and more recent researches have demonstrated that specific CTLs, established by influenza exposure and mostly targeting the virus internal proteins, provide some level of cross-protection against not only antigenically distinct viruses of the same subtype (drift variants) but also different subtypes [10-13]. In vitro testing with T cells isolated from healthy volunteers has been proved that the T cells could cause host cells infected with swine or avian influenza virus to undergo lysis [7,8]. In vivo experiments using a mouse model have also testified the cross-protection offered by influenza T cell responses against lethal challenge with heterologous virus [14,15]. Similar results were obtained in our present study. After exposed to A/PR8(H1N1) virus, mice gained partial protection against lethal A/chicken/Henan/12/2004(H5N1) virus challenge. Four of the total 12 mice survived (Table 2). Transfer of anti-H1N1 antiserum to naive mice could fully protect mice against H1N1 virus challenge, but had no use in defending them against H5N1 virus challenge (Table 1). These indicate that the partial intersubtypic cross-protection mainly relies on the cell-mediated immune responses induced by infection.

Though the cross-protection provided by infection could play a role in alleviating symptoms of H5N1 infection and reducing death, it is after all very limited. Vaccination is an indispensable way to fight against human infection with avian H5H1 virus. Various kinds of vaccines to H5N1 influenza virus have been tried preclinically or clinically, including inactivated whole-virion vaccines [16,17], split vaccines [18,19] and subunit vaccines [20]. However, these vaccines induce only humoral responses and are mainly based on the virus surface protein HA, which are time-consuming on preparation and have been proved to be low immunogenic. Adjuvant addition and increased dose of antigen have to be adopted to increase the immune effect [21,22]. Compared with these conventional vaccines, DNA vaccine has lots of advantages. It induces balanced immune responses and can be prepared in a short time and on a large scale, with high purity and stability [23]. It seems that DNA vaccine is a suitable candidate for pandemic vaccines. According to our previous studies, influenza DNA vaccines based on the surface protein, hemagglutinin or neuraminidase, could provide good protection against lethal challenge with homologous virus, including H5 and other subtypes, whereas those based on the internal protein, either NP or M1, failed to offer satisfactory protection even with multi-dose injection [24-27]. In our present study, after mice had been infected beforehand with A/PR8 (H1N1) to mimic the seasonal influenza virus infection, they were immunized once with H5N1 virus NP DNA, M1 DNA or NP + M1 DNAs, and were then challenged with a lethal dose of the homologous H5N1 virus. The results are somehow unexpected (Table 2). The survival rates offered by a single dose of H5N1 NP DNA or NP + M1 DNA vaccination in pre-exposed mice reached 83% (10/12) and 100% (12/12), respectively. The protective ability (as expressed by survival rate, bodyweight loss and lung virus titer) in these two subgroups of mice had significant difference as compared with that in pre-exposed but unimmunized control group.

Influenza vaccines based on internal proteins induce specific CTL responses that can kill infected cells and help the host recovery from the infection. The antibodies induced by NP or M1 contribute little to providing protective ability, as shown in our and many other researches [23,24,28,29]. In our present study, the level of cellular immune responses, as reflected by the number of the IFN-γ secreting splenocytes in mice, were correlated with degree of protection (Figure 2 and Table 2). A single dose of H5N1 NP DNA or NP + M1 DNAs significantly enhanced the specific cellular response in mice pre-exposed to H1N1 virus, compared with that in the corresponding unexposed mice. In spite of this, we noticed that, though the residue lung virus titers were significantly reduced in pre-exposed mice immunized with NP DNA or NP + M1 DNAs (Table 2), they were not as low as those in mice immunized with HA or NA DNA, as shown in our previous experiments [9]. This may be due to the lack of effective specific Abs to prevent virus from attaching to and releasing among host cells.

The concern about safety of DNA vaccines always exists, including potential integration of plasmid into host genome, induction of autoimmune responses or immunologic tolerance, and so on, but DNA vaccines have been approved to use in animals such as horses [30] and dogs [31]. DNA vaccines have also entered the clinic for initial safety and immunogenicity testing in humans for various infectious diseases, like HIV infections [32], influenza virus infections [33], malaria [34] and hepatitis B infections [35]. All DNA vaccines tested so far were well tolerated with no local or systemic serious adverse effects [36].

## Conclusions

The present study shows that pre-existing immunity against seasonal influenza viruses is useful in offering protection against H5N1 infection, as has been demonstrated before [14]. It also suggests that DNA vaccination may be at least a good choice for individuals innaive to influenza A virus during H5N1 pandemic while strain-matched vaccines are being prepared. Internal protein genes are highly conserved among all influenza A viruses [37]. Whether H5 DNA vaccines encoding the proteins can provide intrasubtypic or even intersubtypic cross-protection in the host pre-exposed to influenza A virus needs to be investigated further.

## Methods

### Viruses and mice

Influenza virus strains used in this study included a mouse-adapted A/PR/8/34(H1N1) virus and an H5N1 virus A/chicken/Henan/12/2004(H5N1), which had been through repeated lung-to-lung passages and adapted in mice as described in our previous studies [25,38]. They were frozen at -70°C until use. All the experiments with live H5N1 virus were performed in a biosafety level 3 containment facilities. SPF female BALB/c mice, aged 6-8 weeks old, were purchased from the Center for Disease Control and Prevention in Hubei Province, China. They were bred and maintained in SPF conditions all along. All the performances on mice in this study followed the Chinese Regulations for the Administration of Laboratory Animals.

### DNA vaccines and peptides

Plasmids pCAGGSP7/NP, pCAGGSP7/M1 were constructed by cloning the PCR products of NP and M1 genes from the A/chicken/Henan/12/2004(H5N1) influenza virus strain into the plasmid expression vector pCAGGSP7, respectively, as described previously [9,24]. The plasmids were propagated in E. coli XL1-blue bacteria and purified using QIAGEN purification kits (QIAGEN-tip 500). The peptide RAVKLYKKLKRE for M1 protein [39] and the peptide TYQRTRALV for NP protein [40], which were used for IFN-γ ELISPOT assay, were synthesized by Shanghai Sangon Biological Engineering Technology & Services Co., Ltd, China.

### Virus infection and challenge

The virus pre-exposure mouse model was achieved by intranasal infection with 5 μl of the viral suspension containing 5LD_50 _influenza virus A/PR/8/34 six weeks before immunization. For challenge experiments, the mice were anesthetized and challenged with 20 μl of the viral suspension containing 20LD_50 _influenza virus A/chicken/Henan/12/2004(H5N1) or A/PR8(H1N1) by intranasal route. The small volume of the virus suspension induced local infection, which was not lethal. On the other hand, the large volume induced total respiratory infection that caused virus shedding from the lung and led to death from viral pneumonia 5 - 10 days later [41].

### Immunization

Mice were immunized with NP DNA, M1 DNA or a mixture of the two DNAs dissolved in 50 μl of Tris-EDTA buffer at a dosage of 50 μg (25 μg each in the mixture of two DNAs) by injection into the quadriceps muscles. After injection, a pair of electrode needles with 5 mm apart was inserted into the muscle to cover the DNA injection site and electric pulses were delivered using an electric pulse generator (Electro Square Porator T830 M; BTX, San Diego, CA). Three pulses of 100 V each, followed by three pulses of the opposite polarity, were delivered to each injection site at a rate of one pulse per second. Each pulse lasted for 50 ms.

### Specimens

Three days after the challenge, six mice from each group were randomly taken out for sample collection. The mice were anaesthetized with chloroform and then bled from the heart with a syringe. The sera were collected from the blood and used for specific IgG Ab assay. After bleeding, the mice were incised ventrally along the median line from the xiphoid process to the point of the chin. The trachea and lungs were taken out and washed 3 times by injecting with a total of 2 ml of PBS containing 0.1% BSA. The bronchoalveolar washes were used for virus titration after removing cellular debris by centrifugation.

### Ab assay by ELISA

The concentrations of IgG Abs against H1N1 virus, NP or M1 protein were measured by ELISA. ELISA was performed sequentially from the solid phase using a series of reagents consisting of first, inactivated H1N1 vaccine, NP or M1 protein prepared by Shanghai Institute of Biological Products; second, serial 2-fold dilutions of sera from each group of immunized or preimmunized mice; third, goat anti-mouse IgG Ab (γ-chain specific) (Southern Biotechnology Associates) conjugated with biotin; fourth, streptavidin conjugated with alkaline phosphatase (Southern Biotechnology Associates); and finally, *p*-nitrophenyl-phosphate. The amount of chromogen produced was measured based on absorbance at 405 - 450 nm in an ELISA reader (Labsystems Multiskan Ascent). Ab-positive cut-off values were set as means + 2 × SD of preimmunized sera. An ELISA Ab titer was expressed as the highest serum dilution giving a positive reaction.

### HI assay

The anti-HA Ab titers were measured by HI assay. Receptor destroying enzyme-treated sera were serially diluted (twofold) in V-shaped 96-well plates. Four hemagglutination units of virus were added to the test and incubated at room temperature for 15 min, followed by addition of 0.5% red blood cells and incubation at room temperature for 30 min. The HI titer is the reciprocal of the highest serum dilution that completely inhibits hemagglutination.

### Passive serum transfer

Naive mice were passively immunized by tail vein injection with 300 μl of pooled serum from mice infected with A/PR8 (H1N1) influenza virus six weeks before or from mice uninfected. One day after the serum transfer, mice were challenged, as described above, with 20LD_50 _of H5N1 or H1N1 influenza virus.

### IFN-γ ELISPOT assay

Spleen cells were isolated from mice for ELISPOT assays at 6 weeks after the vaccination. According to the instruction manual (U-CyTech, Netherlands), 96-well PVDF plates (Millipore, Bedford, MA) were coated with 100 μl of 10 μg/ml rat anti-mouse IFN-γ Ab in PBS and incubated at 4°C overnight. The plates were washed 3 times with sterile PBS and then blocked with 200 μl of blocking solution R and incubated at 37°C for 1 h. Next, 1 × 10^5 ^lymphocytes isolated from the spleen cells were added to the wells in triplicate, stimulated with 2 μg/ml of a synthesized influenza virus peptide, and incubated at 37°C for 18 h. The lymphocytes were then removed, and 100 μl of biotinylated anti-mouse IFN-γ Ab was added to each well and incubated at 37°C for 1 h. Subsequently, 100 μl of properly diluted Streptavidin-HRP conjugate solution was added and incubated at room temperature for 2 h after washing 5 times with PBS. Finally, the plates were treated with 100 μl of AEC substrate solution and incubated at room temperature for 20 min in the dark. The reaction was stopped by washing with dematerialized water. The plates were air-dried at room temperature and read using an ELISPOT reader (Bioreader 4000; Bio-sys, Germany).

### Virus titrations

To examine cytopathic effect, the bronchoalveolar washes, diluted 10-fold serially starting from a dilution of 1:10, were inoculated onto the MDCK cells at 37°C for 2 days. The virus titer of each specimen, expressed as TCID_50_, was calculated by the Reed-Muench method. The virus titer in each experimental group was represented by the mean ± SD of the virus titer per ml of specimens from six mice in each group.

### Statistics

The data from test groups were evaluated by Student's *t*-test; if *P*-value was less than 0.05, the difference was considered significant. The survival rates of mice in test and control groups were compared by using Fisher's exact test.

## List of abbreviations

Ab: antibody; BSA: bovine serum albumin; ELISA: enzyme-linked immunosorbent assay; ELISPOT: enzyme-linked immunospot; HI: hemagglutination inhibition; LD_50_: 50% lethal dose; M1: matrix protein; NP: nucleoprotein; PBS: phosphate buffered saline; SPF: specific pathogen free; TCID_50_: 50% tissue culture infection dose.

## Competing interests

The authors declare that they have no competing interests.

## Authors' contributions

HYC carried out most of the experiments and wrote the manuscript. JW, WJZ and FF did part of the experiment and participated in manuscript preparation. CYH participated in antibody detection and lung virus titration. FYW participated in its design and coordination. ZC was the main designer of the experiment and revised the manuscript. All authors read and approved the final manuscript.

## References

[B1] Tam JS Influenza A (H5N1) in Hong Kong: an overview Vaccine 2002 20 Suppl 2 S77 81 10.1016/S0264-410X(02)00137-8 12110265

[B2] Confirmed Human Cases of Avian Influenza A (H5N1) http://www.who.int/csr/disease/avian_influenza/country/en/16812929

[B3] Cinatl J Jr Michaelis M Doerr HW The threat of avian influenza A (H5N1). Part I: Epidemiologic concerns and virulence determinants Med Microbiol Immunol 2007 196 181 190 10.1007/s00430-007-0042-5 17492465

[B4] Subbarao K Luke C H5N1 viruses and vaccines PLoS Pathog 2007 3 e40 10.1371/journal.ppat.0030040 17335350 PMC1808069

[B5] van Maurik A Sabarth N Dacho HS Bruhl P Schwendinger M Crowe BA Noel Barrett P Kistner O Keith Howard M Seasonal influenza vaccine elicits heterosubtypic immunity against H5N1 that can be further boosted by H5N1 vaccination Vaccine 28 1778 1785 10.1016/j.vaccine.2009.12.008 20018265

[B6] Lee LY Ha do LA Simmons C de Jong MD Chau NV Schumacher R Peng YC McMichael AJ Farrar JJ Smith GL Memory T cells established by seasonal human influenza A infection cross-react with avian influenza A (H5N1) in healthy individuals J Clin Invest 2008 118 3478 3490 18802496 10.1172/JCI32460 PMC2542885

[B7] Jameson J Cruz J Terajima M Ennis FA Human CD8+ and CD4+ T lymphocyte memory to influenza A viruses of swine and avian species J Immunol 1999 162 7578 7583 10358215

[B8] Kreijtz JH de Mutsert G van Baalen CA Fouchier RA Osterhaus AD Rimmelzwaan GF Cross-recognition of avian H5N1 influenza virus by human cytotoxic T-lymphocyte populations directed to human influenza A virus J Virol 2008 82 5161 5166 10.1128/JVI.02694-07 18353950 PMC2395172

[B9] Chen Q Kuang H Wang H Fang F Yang Z Zhang Z Zhang X Chen Z Comparing the ability of a series of viral protein-expressing plasmid DNAs to protect against H5N1 influenza virus Virus Genes 2009 38 30 38 10.1007/s11262-008-0305-2 19067149

[B10] Laddy DJ Yan J Kutzler M Kobasa D Kobinger GP Khan AS Greenhouse J Sardesai NY Draghia-Akli R Weiner DB Heterosubtypic protection against pathogenic human and avian influenza viruses via in vivo electroporation of synthetic consensus DNA antigens PLoS One 2008 3 e2517 10.1371/journal.pone.0002517 18575608 PMC2429965

[B11] Chen Y Qin K Wu WL Li G Zhang J Du H Ng MH Shih JW Peiris JS Guan Y Broad cross-protection against H5N1 avian influenza virus infection by means of monoclonal antibodies that map to conserved viral epitopes J Infect Dis 2009 199 49 58 10.1086/594374 19032063

[B12] Adar Y Singer Y Levi R Tzehoval E Perk S Banet-Noach C Nagar S Arnon R Ben-Yedidia T A universal epitope-based influenza vaccine and its efficacy against H5N1 Vaccine 2009 27 2099 2107 10.1016/j.vaccine.2009.02.011 19356612

[B13] Sui Z Chen Q Wu R Zhang H Zheng M Wang H Chen Z Cross-protection against influenza virus infection by intranasal administration of M2-based vaccine with chitosan as an adjuvant Arch Virol 2010 155 535 544 10.1007/s00705-010-0621-4 20195654

[B14] Kreijtz JH Bodewes R van den Brand JM de Mutsert G Baas C van Amerongen G Fouchier RA Osterhaus AD Rimmelzwaan GF Infection of mice with a human influenza A/H3N2 virus induces protective immunity against lethal infection with influenza A/H5N1 virus Vaccine 2009 27 4983 4989 10.1016/j.vaccine.2009.05.079 19538996

[B15] Tao P Luo M Pan R Ling D Zhou S Tien P Pan Z Enhanced protective immunity against H5N1 influenza virus challenge by vaccination with DNA expressing a chimeric hemagglutinin in combination with an MHC class I-restricted epitope of nucleoprotein in mice Antiviral Res 2009 81 253 260 10.1016/j.antiviral.2008.12.009 19135483

[B16] Vajo Z Wood J Kosa L Szilvasy I Paragh G Pauliny Z Bartha K Visontay I Kis A Jankovics I A single-dose influenza A (H5N1) vaccine safe and immunogenic in adult and elderly patients: an approach to pandemic vaccine development J Virol 2010 84 1237 1242 10.1128/JVI.01894-09 19906909 PMC2812344

[B17] Ikeno D Kimachi K Kino Y Harada S Yoshida K Tochihara S Itamura S Odagiri T Tashiro M Okada K Immunogenicity of an inactivated adjuvanted whole-virion influenza A (H5N1, NIBRG-14) vaccine administered by intramuscular or subcutaneous injection Microbiol Immunol 2010 54 81 88 10.1111/j.1348-0421.2009.00191.x 20377741

[B18] Bresson JL Perronne C Launay O Gerdil C Saville M Wood J Hoschler K Zambon MC Safety and immunogenicity of an inactivated split-virion influenza A/Vietnam/1194/2004 (H5N1) vaccine: phase I randomised trial Lancet 2006 367 1657 1664 10.1016/S0140-6736(06)68656-X 16714186

[B19] Nolan T Richmond PC Formica NT Hoschler K Skeljo MV Stoney T McVernon J Hartel G Sawlwin DC Bennet J Safety and immunogenicity of a prototype adjuvanted inactivated split-virus influenza A (H5N1) vaccine in infants and children Vaccine 2008 26 6383 6391 10.1016/j.vaccine.2008.08.046 18801398

[B20] Treanor JJ Campbell JD Zangwill KM Rowe T Wolff M Safety and immunogenicity of an inactivated subvirion influenza A (H5N1) vaccine N Engl J Med 2006 354 1343 1351 10.1056/NEJMoa055778 16571878

[B21] Stephenson I Nicholson KG Colegate A Podda A Wood J Ypma E Zambon M Boosting immunity to influenza H5N1 with MF59-adjuvanted H5N3 A/Duck/Singapore/97 vaccine in a primed human population Vaccine 2003 21 1687 1693 10.1016/S0264-410X(02)00632-1 12639491

[B22] Hehme N Engelmann H Kuenzel W Neumeier E Saenger R Immunogenicity of a monovalent, aluminum-adjuvanted influenza whole virus vaccine for pandemic use Virus Res 2004 103 163 171 10.1016/j.virusres.2004.02.029 15163505

[B23] Epstein SL Tumpey TM Misplon JA Lo CY Cooper LA Subbarao K Renshaw M Sambhara S Katz JM DNA vaccine expressing conserved influenza virus proteins protective against H5N1 challenge infection in mice Emerg Infect Dis 2002 8 796 801 12141964 10.3201/eid0808.010476 PMC2732511

[B24] Chen Z Sahashi Y Matsuo K Asanuma H Takahashi H Iwasaki T Suzuki Y Aizawa C Kurata T Tamura S Comparison of the ability of viral protein-expressing plasmid DNAs to protect against influenza Vaccine 1998 16 1544 1549 10.1016/S0264-410X(98)00043-7 9711802

[B25] Qiu M Fang F Chen Y Wang H Chen Q Chang H Wang F Zhang R Chen Z Protection against avian influenza H9N2 virus challenge by immunization with hemagglutinin- or neuraminidase-expressing DNA in BALB/c mice Biochem Biophys Res Commun 2006 343 1124 1131 10.1016/j.bbrc.2006.03.088 16580631

[B26] Chen Z Kadowaki S Hagiwara Y Yoshikawa T Matsuo K Kurata T Tamura S Cross-protection against a lethal influenza virus infection by DNA vaccine to neuraminidase Vaccine 2000 18 3214 3222 10.1016/S0264-410X(00)00149-3 10869766

[B27] Fang F Cai XQ Chang HY Wang HD Yang ZD Chen Z Protection abilities of influenza B virus DNA vaccines expressing hemagglutinin, neuraminidase, or both in mice Acta Virol 2008 52 107 112 18564897

[B28] Chen Z Yoshikawa T Kadowaki S Hagiwara Y Matsuo K Asanuma H Aizawa C Kurata T Tamura S Protection and antibody responses in different strains of mouse immunized with plasmid DNAs encoding influenza virus haemagglutinin, neuraminidase and nucleoprotein J Gen Virol 1999 80 Pt 10 2559 2564 10573147 10.1099/0022-1317-80-10-2559

[B29] Epstein SL Kong WP Misplon JA Lo CY Tumpey TM Xu L Nabel GJ Protection against multiple influenza A subtypes by vaccination with highly conserved nucleoprotein Vaccine 2005 23 5404 5410 10.1016/j.vaccine.2005.04.047 16011865

[B30] CDC and Fort Dodge Animal Health Achieve First Licensed DNA Vaccine http://www.cdc.gov/media/pressrel/r050718.htm

[B31] Elvidge S Melanoma vaccine for dogs Nature biotechnology 2010 28 189 10.1038/nbt0310-189a20212469

[B32] MacGregor RR Boyer JD Ugen KE Lacy KE Gluckman SJ Bagarazzi ML Chattergoon MA Baine Y Higgins TJ Ciccarelli RB First human trial of a DNA-based vaccine for treatment of human immunodeficiency virus type 1 infection: safety and host response J Infect Dis 1998 178 92 100 9652427 10.1086/515613

[B33] Smith LR Wloch MK Ye M Reyes LR Boutsaboualoy S Dunne CE Chaplin JA Rusalov D Rolland AP Fisher CL Phase 1 clinical trials of the safety and immunogenicity of adjuvanted plasmid DNA vaccines encoding influenza A virus H5 hemagglutinin Vaccine 2010 28 2565 2572 10.1016/j.vaccine.2010.01.029 20117262

[B34] Le TP Coonan KM Hedstrom RC Charoenvit Y Sedegah M Epstein JE Kumar S Wang R Doolan DL Maguire JD Safety, tolerability and humoral immune responses after intramuscular administration of a malaria DNA vaccine to healthy adult volunteers Vaccine 2000 18 1893 1901 10.1016/S0264-410X(99)00407-7 10699338

[B35] Rottinghaus ST Poland GA Jacobson RM Barr LJ Roy MJ Hepatitis B DNA vaccine induces protective antibody responses in human non-responders to conventional vaccination Vaccine 2003 21 4604 4608 10.1016/S0264-410X(03)00447-X 14575774

[B36] Schalk JA Mooi FR Berbers GA van Aerts LA Ovelgonne H Kimman TG Preclinical and clinical safety studies on DNA vaccines Hum Vaccin 2006 2 45 53 17012886 10.4161/hv.2.2.2620

[B37] Suarez DL Evolution of avian influenza viruses Vet Microbiol 2000 74 15 27 10.1016/S0378-1135(00)00161-9 10799775

[B38] Wu J Zhang F Fang F Chang H Wang F Yang Z Sun B Chen Z Efficacy of inactivated vaccine against H5N1 influenza virus infection in mice with type 1 diabetes Vaccine 28 2775 2781 10.1016/j.vaccine.2010.01.037 20117261

[B39] Watabe S Xin KQ Ihata A Liu LJ Honsho A Aoki I Hamajima K Wahren B Okuda K Protection against influenza virus challenge by topical application of influenza DNA vaccine Vaccine 2001 19 4434 4444 10.1016/S0264-410X(01)00194-3 11483269

[B40] Saha S Yoshida S Ohba K Matsui K Matsuda T Takeshita F Umeda K Tamura Y Okuda K Klinman D Xin KQ A fused gene of nucleoprotein (NP) and herpes simplex virus genes (VP22) induces highly protective immunity against different subtypes of influenza virus Virology 2006 354 48 57 10.1016/j.virol.2006.04.015 16945400

[B41] Hagiwara Y Komase K Chen Z Matsuo K Suzuki Y Aizawa C Kurata T Tamura S Mutants of cholera toxin as an effective and safe adjuvant for nasal influenza vaccine Vaccine 1999 17 2918 2926 10.1016/S0264-410X(99)00135-8 10438064

